# Assessment of the Effect of Cleanliness on the Visual Inspection of Aircraft Engine Blades: An Eye Tracking Study

**DOI:** 10.3390/s21186135

**Published:** 2021-09-13

**Authors:** Jonas Aust, Antonija Mitrovic, Dirk Pons

**Affiliations:** 1 Department of Mechanical Engineering, University of Canterbury, Christchurch 8041, New Zealand; dirk.pons@canterbury.ac.nz; 2 Department of Computer Science and Software Engineering, University of Canterbury, Christchurch 8041, New Zealand; tanja.mitrovic@canterbury.ac.nz

**Keywords:** eye tracking, inspection, visual search strategy, decision making, MRO, aircraft engine maintenance, blade inspection, visual perception, attentional trajectory

## Abstract

Background—The visual inspection of aircraft parts such as engine blades is crucial to ensure safe aircraft operation. There is a need to understand the reliability of such inspections and the factors that affect the results. In this study, the factor ‘cleanliness’ was analysed among other factors. Method—Fifty industry practitioners of three expertise levels inspected 24 images of parts with a variety of defects in clean and dirty conditions, resulting in a total of N = 1200 observations. The data were analysed statistically to evaluate the relationships between cleanliness and inspection performance. Eye tracking was applied to understand the search strategies of different levels of expertise for various part conditions. Results—The results show an inspection accuracy of 86.8% and 66.8% for clean and dirty blades, respectively. The statistical analysis showed that cleanliness and defect type influenced the inspection accuracy, while expertise was surprisingly not a significant factor. In contrast, inspection time was affected by expertise along with other factors, including cleanliness, defect type and visual acuity. Eye tracking revealed that inspectors (experts) apply a more structured and systematic search with less fixations and revisits compared to other groups. Conclusions—Cleaning prior to inspection leads to better results. Eye tracking revealed that inspectors used an underlying search strategy characterised by edge detection and differentiation between surface deposits and other types of damage, which contributed to better performance.

## 1. Introduction

### 1.1. Industrial Context

In the aviation industry, engine maintenance, repair and overhaul (MRO) is essential to ensure the continued airworthiness of aircrafts and safe flight operations. Engines are inspected on a frequent basis, either after a certain amount of flight hours or cycles (planned shop visit), or after an unexpected event such as a bird strike or flying through volcanic ash (unplanned shop visit) [1]. The inspection is predominately performed by human operators and thus there is an inherent risk of human error [2,3,4,5]. According to the International Air Transport Association (IATA), one of the top three causes for aircraft accidents are maintenance and inspection errors, and every third accident chain started with an event caused by incorrect maintenance [6,7]. A Federal Aviation Authority (FAA) report on aircraft maintenance risks concluded that maintenance errors contributed to 27.4% of fatalities and 6.8% of incidents [6]. The most common component failures occur on the engine. Compressor and turbine blades are the most rejected parts during engine maintenance [8], since they are exposed to extreme operating conditions, including high centrifugal forces, high pressures, high temperatures (turbine section), foreign object damage (FOD in the compressor section) and vibrations [9,10,11,12]. Hence, the frequent inspection of those parts is of high importance in order to find any damage before it propagates.

### 1.2. Engine Blade Inspection

**Figure 1:**
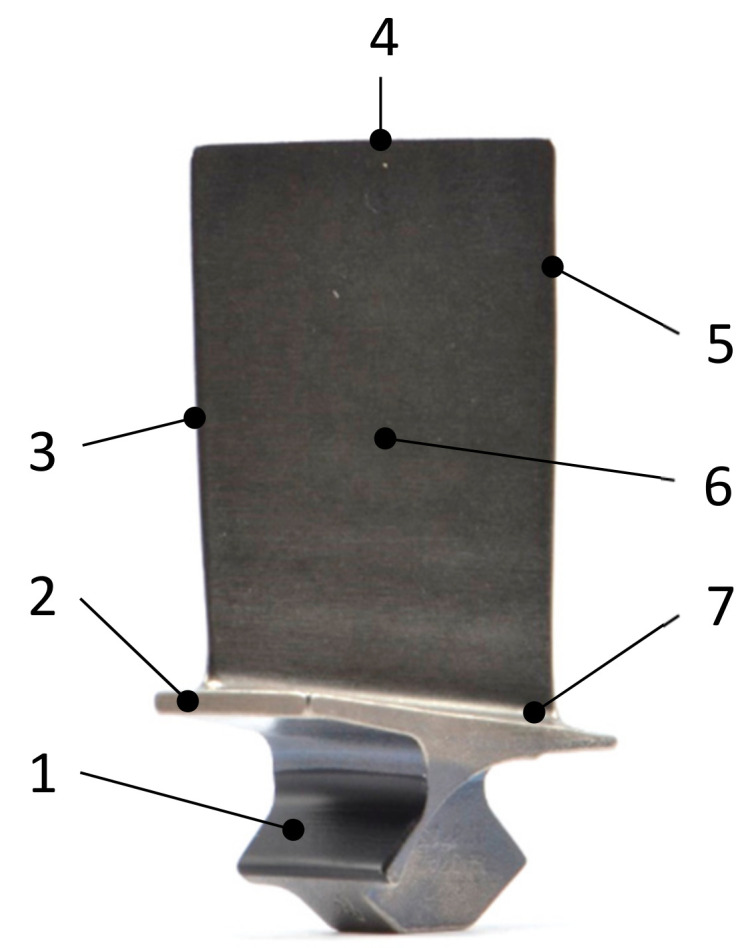
High-pressure compressor (HPC) blade with highlighted blade regions: (1) blade root; (2) platform; (3) trailing edge; (4) blade tip; (5) leading edge; (6) airfoil; (7) platform radius.

Visual inspection is the most common non-destructive testing (NDT) technique used and accounts for approximately 90% of inspections of aircraft parts, including engine blades [2,13,14]. Several areas of the blade (identified in Figure 1) have to be inspected for different types of defects. There are two possible inspection errors that can occur, i.e., when a non-defective blade is incorrectly identified as defective (false positive), and when a defective blade is incorrectly classified as serviceable (false negative). While false positives have no negative impact on flight safety, they directly increase the maintenance cost. A non-defective blade that is classified as defective is subsequently repaired or scrapped, which introduces additional costs for unnecessary labour and material [15]. In contrast, missing a blade defect can cause severe damage to the engine and fuselage of the aircraft, with the potential to harm passengers and even lead to fatalities [16]. This may be caused by improper maintenance and inspection, which is prone to human error tendencies as well as lack of accuracy, reliability, subjectivity, consistency and repeatability [3,17,18].

One factor that may affect the detectability of defects is the cleanliness. There are two conditions of part cleanliness. When the engine is first inspected, the blades are dirty and often covered with deposits. After disassembly and repair, the blades are in a clean condition (high-pressure water sprayed and alkaline bathed) and have to be re-inspected before installation back on the engine.

There is a need to determine the effect of part cleanliness on the inspection results. This is important for the industry in regard to future improvement, e.g., to determine what cleaning processes prior to inspection could improve the inspection performance. Counterintuitively, it is also possible that cleaning might make detection poorer, e.g., there is some anecdotal evidence that deposits might highlight defects. This paper applies eye tracking and statistical testing to evaluate the relationships between cleanliness and detectability, for the specific case of engine blades. The results show that clean parts are better than dirty ones in relation to inspection performance.

## 2. Literature Review

### 2.1. Research on Visual Inspection

During engine maintenance, blades are checked at several stages in the process, in different conditions and for different types of damages [19]. The first inspection is by borescopic means, whereby an inspector looks for any operational damage. If a defect is found that validates a tear down, subsequent module and piece-part inspections are performed. The parts are generally in dirty conditions, although a compressor wash may precede. Damaged parts are either scrapped or repaired depending on the severity, location and type of defect. After repair, the parts are visually inspected as part of the quality assurance processes to ensure that the damage was successfully repaired and no repair limits are exceeded. During reassembly of the engine, the blades are inspected again for any transportation or handling damages that might have occurred between the repair shop and the assembly line.

#### 2.1.1. Parameters Affecting Visual Inspection

The FAA published a list of factors affecting visual inspection, namely inspection personnel qualifications and training, inspection area access, lighting, pre-cleaning and the working environment [20]. In [15], additional factors impacting the inspection performance were gathered, which were grouped into task-related, individual, environmental, organisational and social factors. More recently, Aust and Pons [21,22] applied 6 M categorisation to group impact factors that affect borescope inspection. While the importance of those factors is generally accepted, it remains difficult to quantify them. Few studies quantitatively assessed the effect of some factors on dent and crack detection in composite panels [23,24,25,26]. The assessed factors included part-related and work-environmental parameters, including surface colour and finish, part cleanliness, inspection distance and angle, and lighting. Furthermore, the effect of personal factors such as professional qualification, training, experience, education, visual capability, age and gender was evaluated. The cleanliness factor, however, was only analysed based on artificially created dirt using soot and coffee powder [24].

While those factors were analysed for the inspection of composites, the effect on blade inspection might be changed due to several significant differences, including the operating environment, complex part geometry, materials, surface finishes and defect types [19].

When it comes to metal parts, a study by See [27] analysed similar factors for the inspection of precision manufactured parts. However, the defect types analysed were mostly welding defects such as creases, cuts or puffiness, and the parts were in new and clean condition.

#### 2.1.2. Inspection Performance

The assessment of different inspection tasks, such as the inspection of subsea structures and pipelines [28], piston rings [29], highway bridges [30], acoustical tiles [31] and precision manufactured parts for nuclear weapons [27], showed detection accuracies of 53%, 67%, 52%, 76%, and 75%, respectively. Studies on inspection accuracies within the aviation domain presented detection rates of 68% for the visual inspection of aircraft fuselage [32], 42% in cargo bay inspection [33], and 57–98% for the magnetic particle inspection of landing gear components [34]. The main focus in this industry is on crack and dent detection, predominantly in composite materials, and thus inspection results are commonly reported as probability of detection (PoD) curves, i.e., the chance of defect detection depending on the defect size [23,24,25,26,35].

### 2.2. Eye Tracking

Eye tracking is a non-invasive method using near-infrared light and cameras to record the position and movement of the eye in the form of gaze points (x/y-coordinates), fixations (dwell times), fixation sequence (gaze paths, also referred to as the attentional trajectory), number of fixations and saccades [36]. Monitoring may be monocular or binocular, screen-based or through wearable glasses [37,38]. The technology has improved in accuracy and usefulness [39,40,41], enabling widespread application. Examples of areas of application are health and safety [36], retail [42,43], medical [44,45,46], education [47,48,49], neuroscience and psychology [50,51], marketing and advertisement [52], conservation and animal welfare [53], construction [54], automotive [55], aerospace [54,56], maritime [54], consumer electronics [57,58], tourism [59] and security [60]. Eye tracking helps illuminate the visual processes of perception [36,61], task-specific human behaviour [62] and cognitive processing during complex tasks [62,63]. It is also useful for training purposes [64,65] to assess proficiency [51] and compare levels of expertise [36].

A closer look at the aviation industry revealed that eye tracking was predominantly used in flight simulators to access and monitor pilot performance in different situations [56,66], such as flying in challenging conditions [67,68], landing [69,70], navigating [71] and the failure of cockpit instruments [72]. Most of these studies compared the performance of expert pilots with learners. Other research tested the usability and effects of new cockpit instruments [73,74] and changed cockpit layouts [75]. An interesting work in that area is the attempt to use eye tracking as an input to control the aircraft [76]. Pilot training is another well-studied area in eye tracking research [77,78,79]. Only a few studies applied eye tracking to scanning and decision-making tasks outside the cockpit, including air-traffic control [80,81] and airport luggage screening [82,83,84].

The two main areas in which eye tracking has been used to explore visual search and diagnostics tasks are the medical [85] and manufacturing industries [86]. While in healthcare, the ‘inspection’ is related to the condition assessment of living beings, in the production industry, it refers to the inspection of parts. The assessment of the human body can be considered as being more complex than the quality assessment of manufactured parts, since no human or medical condition is identical. Nonetheless, they involve a similar procedure, including task initiation and access of the area to be assessed, followed by a systematic search for any anomalies or alarming conditions, and a detailed examination [87]. Subsequently, a decision as to whether this condition is critical and how it must be treated has to be made. Due to the similar procedure, the findings of one research area might be interesting for the other.

Sometimes, the inspection and decision can be made on the actual (body) part, while other times, it must be made based on images or videos due to restricted accessibility, e.g., borescope inspection in an industrial context, or X-ray, mammography or CT scan in healthcare [88,89,90,91]. When it comes to inspections as part of the quality management system in manufacturing, eye tracking was applied to evaluate the defect detection of sheet metal [92,93], porcelain plates [86], empty bottles [94], woven fabrics [86], integrated circuit chips [95], tapered roller bearings [86], tin cans [86] and electrical edge connectors [86].

Only one research project was found that applied eye tracking to inspection within the aviation maintenance domain. Those researchers published several papers on the development and evaluation of advanced eye tracking technology, using a virtual reality (VR) eye tracker for inspection training in a three-dimensional aircraft inspection simulator [96,97,98,99]. The inspection task involved the search for damaged conduits, cracks and corrosion in an aircraft cargo space. The effect of cognitive feedback from the eye tracker on inspection training improvements was assessed [96]. A decrease in fixations and search time was presumed to indicate an improved visual search strategy. While the authors of the earlier publications stated that the detection accuracy (number of detected and missed defects) was another metric used to measure the effect of training, the results were not reported [96]. Only a later publication reported on the performance of novices before and after training, with inspection accuracies of 13.77% and 42.27%, respectively [33].

### 2.3. Gaps in the Body of Knowledge

The inspection performance for operational defects such as foreign object damage (FOD) on engine components has yet not been quantified. The inspection of such components is challenging due to the dirty part condition. Previous attempts were made to assess the cleanliness effect in composite panel inspection [24,25]. However, the material shows different types of defects that were introduced post hoc and manually by the researcher [24]. Furthermore, the cleanliness factor was only analysed based on artificially created dirt using soot and coffee powder. None of the previous studies applied eye tracking to analyse the underlying cognitive and attentional processes. Only one research group used eye tracking for inspection training for aircraft cargo bays in a virtual maintenance environment [33,96]. However, no personal, environmental or stimuli-related factors were addressed. Moreover, no work was found that assessed the visual inspection task of metal parts with complex geometries such as engine blades and the impact factors affecting the inspection task, e.g., cleanliness.

## 3. Materials and Methods

### 3.1. Research Objective and Methodology

The objective of this study was to examine the relationship between cleanliness and the detectability of defects. We were particularly interested in how this is moderated by operator demographics (e.g., expertise), and whether this changed for various types of defects. The area under examination was compressor blades from the V2500 jet engine.

Photographs were taken of clean and dirty blades from a variety of engines with different defects (including non-defective blades). Images were shown to industry practitioners under eye tracking observation. The results were analysed statistically and qualitatively.

### 3.2. Research Sample (Stimuli)

The parts under examination were N = 12 high-pressure compressor (HPC) blades of various engines from different airlines. HPC blades were chosen as they are close to the engine intake and thus exposed to air containments and FOD. The different operating environments lead to different blade conditions ranging from lightly to heavily dirty and various types of deposits. The sample size of this research comprised twelve blades. Of those twelve blades, four were non-defective and eight were defective. The blades covered the most common types of defects including nicks, dents, and tears. All blades were removed from service and scrapped. The parts were mutilated to remove serial numbers. This did not affect the airfoil area under inspection. Of each part, two photographs were taken—one before cleaning the part, i.e., in dirty condition, and one after cleaning. This led to 24 images that were presented to 50 participants, resulting in a total of N = 1200 observations. The large dataset lends itself to statistical analysis.

The image acquisition was a two-stage process. First, images of the blades in dirty condition were taken before the parts underwent a cleaning procedure. Subsequently, a second set of images was taken of the now cleaned blades with the same camera setup and settings. For the image acquisition, we used the same set-up as in [100]. This comprised a self-made light tent and three LED ring-lights (LSY 6W manufactured by Superlux, Auckland, New Zealand) placed on the left and right side and on the top of the light tent for optimal illumination. A Nikon D5200 DSLR camera with Nikkor Macro lenses with a focal length of 105 mm (both manufactured by Nikon Corporation, Tokyo, Japan) captured the images with a resolution of 24.1 mega pixels in JPEG format.

**Figure 2:**
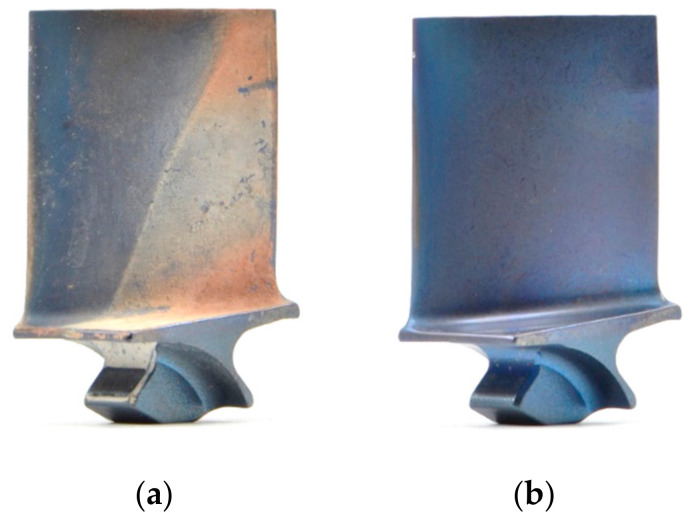
(**a**) Dirty blade before cleaning; (**b**) blade after cleaning.

After images of the blades in dirty condition were acquired, the blades went through a cleaning process. First, high-pressure water spraying was used to remove loose or powdery soil. Subsequently, the parts were soaked in an alkaline rust remover solution and dried. A sample blade before and after cleaning is presented in Figure 2 below.

### 3.3. Research Population

We recruited N = 54 participants, 3 female and 51 male $\left(Mean_{Age}=44.5;\;Standard\;Deviation_{Age}=10.33\;years\right)$ from our industry partner, a maintenance repair and overhaul (MRO) shop for the V2500 aircraft engine. To compensate the technical limitations of the eye tracking device [101] and avoid loss of data, exclusion criteria included any neurological disorder, impaired vision, eye surgery, photochromic glasses or varifocals, eye movement or alignment abnormalities, long and thick eyelashes, and excessive makeup.

From the staff meeting those criteria, the research population was purposely selected in a way that meant different levels of expertise and experience were covered. This was determined based on their job assignment and the number of years spent working in the aviation industry, in particular in the field of blade inspection. The participants were divided into three groups of 18 participants: (a) on-bench and borescope inspectors, hereinafter referred to as inspectors, (b) power plant and production engineers referred to as engineers and (c) aircraft tradespersons, from now on referred to as assembly operators. Participants had worked between 18 months and 35 years in the aviation industry $\left(M_{Experience}=17.7;\;SD_{Experience}=9.4\;\mathrm{years}\right)$.

Four participants (two engineers and two assembly operators) were excluded from the studies, as the eye tracker was unable to record sufficient gaze data of the participants to meet the minimum data quality standard (eye recognition rate > 85%). This could have been caused by, e.g., extensive blinking, some issues with their glasses, or facemasks covering their eyes. Thus, when referring to the research population in the following sections, it only refers to the 50 participants that met the requirements and where the eye tracking data could be used for further analysis.

Before the actual eye tracking task began, some obligatory tasks had to be completed, including filling out a questionnaire, providing informed consent, task introduction, participant positioning and calibration of the eye tracker. The questionnaire was used to obtain information about the participants’ demographics, pre-existing knowledge on visual inspection, in particular of blade, as well as previous work experience in the aviation industry. An overview of the participants’ demographics can be found in Table 1. This information was used to group the participants into three skill levels. We also asked for any medical condition, such as eye surgery, to ensure that the person could participate without any risks. The participants were introduced to the task and informed about their right to withdraw from the study at any time. Consent was obtained, which allowed for the collection of their individual data. The participants volunteered and received no compensation for their time. All experimental procedures and materials were approved by the by the Human Ethics Committee of the University of Canterbury (HEC 2020/08/LR-PS).

### 3.4. Eye Tracking Approach

#### 3.4.1. Technology Setup

For this study, a Tobii Pro Spectrum (manufactured by Tobii AB, Danderyd, Sweden) eye tracker was used to capture the participants’ eye movement. The device has a sampling rate of 300 Hz, an accuracy of 0.3° and a precision of 0.06° at optimal conditions, i.e., at a distance of 65 cm [102]. Two cameras (one per eye) captured stereo images of both eyes of the participants (binocular). The average gaze sampling rate was 93.3% (SD 6.1%).

**Figure 3:**
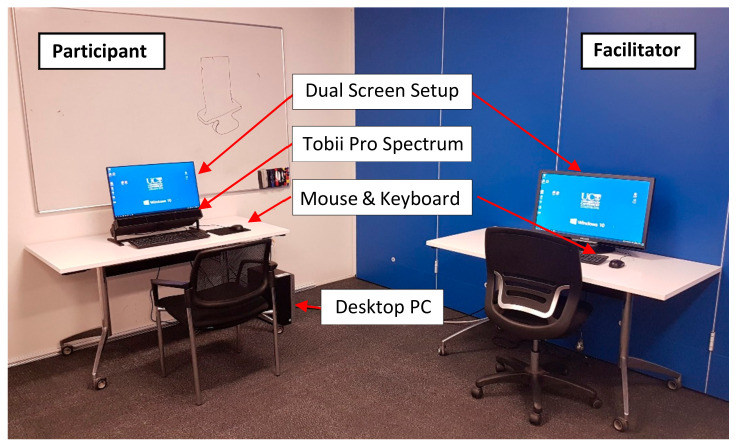
Eye tracking setup with dual monitor for real-time assessment.

In addition to the Tobii Pro Spectrum, the eye tracking setup consisted of a desktop computer (HP Elitedesk with Intel i7 3.4 GHz processor and 16 GB RAM) with Windows 10 Enterprise operating system, a standard cable keyboard and a laser mouse (Figure 3). For the presentation of the images, we used the 24.8-inch built-in LED screen (EIZO FlexScan EV2451) of the eye tracker with a resolution of 1920 × 1080 pixels. The monitor was mounted on top of the eye tracker that was attached to a stand (desktop mount). Equipment of the University of Canterbury was used.

A dual screen setup was chosen for several reasons. First, it was easier for the facilitator launching Tobii Pro Lab, opening the PowerPoint presentation, starting the calibration and storing the collected data. Additionally, it allowed one to monitor the participants’ eye movements in real time and take notes during the study. Most important was the compliance with the COVID-19 pandemic health and safety measures such as social distancing. The study complied with all health and safety regulations of our industry partner and followed the manufacturer’s ‘health and safety recommendations for data collection during the COVID-19 pandemic’, which is in accordance with the U.S. Center for Disease Control and Prevention (CDC) and Occupational Safety and Health Administration (OSHA) [103]. Facemasks did not affect the eye tracking results as long as the masks did not cover the line of sight between the participants’ eyes and the eye tracker [104].

#### 3.4.2. Stimuli Presentation

The images were presented in a PowerPoint presentation, whereby the participants could navigate through the presentation at their own speed. For this research, the time each participant had to perform the inspection was not limited, since in practice, the accuracy of the safety critical inspection task is more important than the speed. However, participants could not go back to review an image. The presentation was created with PowerPoint 2016 version 16.0.4266.1001 (developed by Microsoft, Redmond, WA, USA). The main benefit of PowerPoint is the ‘pen tool’ [105]. This function allowed the participants to mark any defects they found, which represents the real situation with the exception that the marking is not physically on the blade but digitally saved on the image. This enabled the collection of additional information about the detections and location of the findings.

A fundamental rule of eye tracking is that the stimuli should be represented as close as possible to the real-world situation which is trying to be assessed. This will make the findings more applicable and allows for generalisation and valid conclusions [106]. For piece-part inspection, the engine manual permits the use of threefold to tenfold magnification as an aid to examine any condition found. For this reason and due to the relatively large distance between the participants’ eyes and the computer screen that should be preserved at all times, we decided to present the parts with a threefold magnification. The resulting image sizes on the screen measured 975 by 645 pixels, or 24 by 15.9 cm. This corresponds to 39.8° by 26.4° visual angle. All images were presented in the centre of the screen.

The images were presented in random order in terms of stage numbers, defect types, non-defective and defective, and dirty and cleaned parts. The dataset presented in this paper comprises 24 images and is a subset of a bigger study comprising 120 images. Due to the size and variety of the dataset, it was assumed that memory effects did not occur, i.e., participants were not able to recognise the same blade in dirty and clean condition. This was proven in another sub-study, results not reported here, whereby the exact same images of the same parts and under same conditions was shown to the participants twice. A high variation in the participants’ detection performance indicated that they did not remember seeing the picture before, nor their previous inspection decision for that blade.

### 3.5. Data Collection

First, the eye tracking device was positioned 65 cm in front of the participants and adjusted to their height. This was followed by a calibration of the eye tracker. After a successful calibration, the main inspection task began. The images were shown to the participants in random order, and they were asked to search for and locate any defects that could warrant an engine tear down and scrapping or repairing of the blade. When such a defect was found, the participants were instructed to draw a circle around it. Once the participants were confident that they found all defects on a blade, they could advance to the next image. The participants had as much time as they needed, but could not go back and revisit an image. An exit survey was conducted which asked what the participants found difficult, whether they had any particular search approach, and how confident they were with their performance.

### 3.6. Determination of Ground Truth

The acquired piece-part images were presented to two bench-inspection experts who routinely inspect blades. The first one was asked to identify and mark all safety critical defects. The second inspection expert was then asked to confirm this determination. In case of a deviation between the two, the actual part and the engine manual was consulted, and the defects were measured before a final decision was made. This formed the ground truth against which the individual inspection results of the participants were compared later on. After determining the ground truth, the two inspection experts were excluded from the subsequent tasks.

### 3.7. Data Analysis

The data were analysed to test Hypotheses H1 and H2 (Table 2). The independent variables were the cleanliness of the blade, defect type, participants’ level of expertise, work experience, previous experience in inspection, education, visual acuity, and confidence rating. The dependent variables included inspection accuracy, inspection time, and search pattern. All variables were statistically analysed using TIBCO Statistica, version 13.3.0 (developed by TIBCO, Palo Alto, CA, USA).

The inspection results (decision) of each participant were extracted from the eye tracking data and PowerPoint presentations and compared to the ground truth to determine whether their decision was correct or incorrect. The four possible inspection outcomes are true positive (TP), false positive (FP), true negative (TN) and false negative (FN). An overview of all metrics used in this study can be found in Table 3. The primary output metrics selected for reporting in this paper were *Inspection Accuracy* and *Inspection Time*.

The Tobii Pro Spectrum collected a variety of data on eye movement, including gaze points, fixations (dwell times), the number of fixations and saccades. Additionally, participant actions such as mouse clicks and key presses were recorded. Tobii Pro Lab software version 1.145 [107] was used for the subsequent analysis of the collected data and the creation of additional metrics, such as times of interest (TOIs).

To visualise the gaze movements of the participants, we used the fixations in defined TOIs to generate heat maps and gaze plots. The latter enabled us to analyse the search patterns and to understand whether there was a correlation between the search strategy and the performance of the participants.

Areas of interest (AOIs) were not used in this study due to the small size of the defect and in consideration of the accuracy and precision of the eye tracking hardware, which would have resulted in high selectivity with the risk of not collecting meaningful data. The manufacturer proposed the ‘1 degree’ guideline, i.e., the AOI shall not be smaller than one degree visual angle, which translates to 50 × 50 pixel on the computer screen [108,109]. Others reported that AOIs smaller than 10% of the stimuli height and width or smaller than 200 × 200 pixels should be avoided [110,111]. Rather than use AOIs, the researchers instead examined the gaze plots and heat maps around each defect to determine how much attention it was given. The time aspect was determined by the heat maps (intensity of colour), and quantitatively measured as the average time per image.

## 4. Statistical Results

There are two dependent variables of interest for inspection performance, namely *Inspection Accuracy* and *Inspection Time*. These are each analysed in turn.

### 4.1. Inspection Accuracy

The inspection accuracy describes the ratio of correct decisions (true positives and true negatives) divided by the number of blades and is presented as a percentage. The inspection accuracies of the different expertise groups are presented in Table 4 for dirty and clean blades. The average improvement describes the percentage change from dirty to clean blades to demonstrate the direction and size of the effect. This was calculated for each participant first, and then the mean and standard deviation for each expertise group were formed.

The results indicate that on average, the performance improved for all three groups of expertise, with engineers improving the most and assembly operators the least.

#### 4.1.1. Hypothesis Testing

**Hypothesis** **H1a.** *Inspectors perform better in terms of inspection accuracy than non-inspecting staff.*

This hypothesis was tested by generalised linear/non-linear Logit testing with *Expertise* as the categorical variable, and *Decision* as the dependent variable. The statistical analysis (Table 5) shows that *Expertise* is not a significant variable for *Inspection Accuracy* (*Decision*) with photographed defects and unlimited inspection time. However, it shoud be noted that *Expertise* was found to have a significant correlation with *Inspection Time*, as shown later in Section 4.2.

The finding that trained inspectors do not perform significantly better than assembly operators or engineer is surprising. This might be due to the somewhat new task and environment: while inspectors perform visual checks on a daily basis, they would normally hold the part in their hands as opposed to making a serviceability decision based on an image. The latter only applies to borescope inspectors (N = 2). Engineers and assembly operators in turn are not assigned to inspection tasks, and thus the situation was new for them, especially for assembly operators. Another explanation for the non-significance of the level of expertise on the decision might be that inspectors become complacent, while non-inspecting staff are not familiar and thus perform the task slower, but potentially in a more detailed and rather cautious manner (see Section 4.1.2). It might also show the best achievable inspection performance of humans in general, using solely their vision.

**Hypothesis** **H2a.** *Cleaned blades lead to improved inspection performance measured as inspection accuracy compared to blades in dirty condition.*

This hypothesis was also analysed by generalised linear/non-linear Logit testing, with *Cleanliness* and *Decision* being the categorical and dependent variable, respectively. The results shown in Table 6 highlight a significant correlation between the part cleanliness and decision of the part serviceability. The odds ratio shows that detecting a defect on cleaned blades is over three times more likely than detecting the same defect on dirty blades. The comparable one-way ANOVA result is F(1, 1198) = 68.455, *p* < 0.001.

Although this might be self-explanatory, it should be noted that for ‘clean’ blades, all the pre-existing deposits were removed in the cleaning process. The results support a conclusion that clean blades provided fewer distracting features, and no deposits obscured the defect, thereby leading to higher inspection accuracy. The assumption that deposits highlight the defect and thus improve the detectability was not supported by this study.

#### 4.1.2. Statistical Model for Inspection Accuracy

The situation is more complex than the simple hypothesis test implies, because there are other factors that may affect the inspection performance. Thus, a statistical model was constructed around the *Inspection Accuracy*. The variable *Decision* takes values of 1 (correct decision) or 0 (incorrect decision). Hence, an appropriate statistical analysis is Logit Odds Ratio. A statistical model was built with *Inspection Accuracy* (*Decision*) as the dependent variable. The categorical factors were *Expertise*, *Visual Acuity*, *Education*, *Previous Inspection Experience*, *Cleanliness*, *Defect Type*, and *Confidence Rating*. The continuous predictors were *Work Experience* and *Inspection Time*. Due to the uneven distribution of male (N = 47) and female (N = 3) participants, a statistical analysis of gender was not performed. It should be noted that the following variables are demographic parameters of the participants and not recorded at the level of individual blades: *Work Experience*; *Confidence Rating*; *Expertise*; *Visual Acuity*; *Education*; and *Previous Inspection Experience*. Additionally, a test of all effects was performed. The results are shown in Table 7.

**Figure 4:**
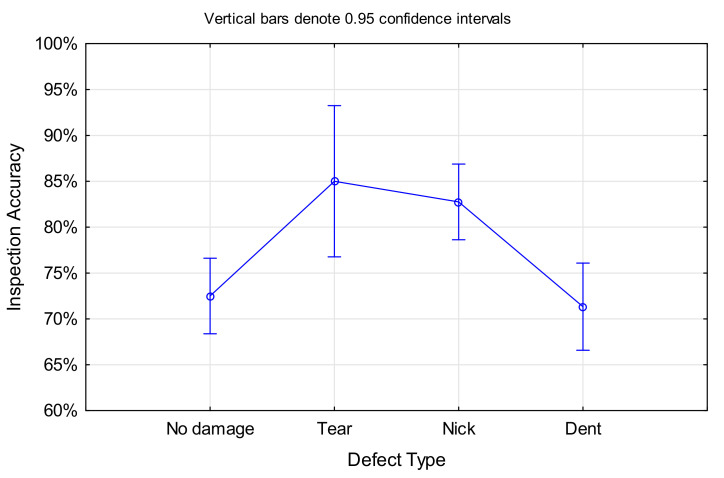
Effect of defect type on inspection accuracy.

Apart from *Cleanliness* (see Section 4.1.1), the only other factor that was significant for the *Inspection Accuracy* was the *Defect Type*, F(3, 1196) = 7.0352, *p* < 0.001 (ANOVA results in Figure 4, please note that this ignores cleanliness as a factor). However, not all defect types were equally detectable. Nicks and tears showed a higher chance of detection compared to dents, independent of the blade cleanliness. Furthermore, participants generally showed a lower performance on non-defective blades, with no significant difference between clean and dirty blade condition.

The unforeseen finding is that all demographic variables that are somewhat related to the skill level of the participants, including *Expertise*, *Work Experience*, *Previous Inspection Experience* and *Education*, had no significant effect on the inspection outcome. In addition to the before-mentioned reasons, this could stem from the nature of the industry, whereby all employees are aware of the negative consequence that a missed defect might have.

Other non-significant factors were *Inspection Time, Confidence Rating* and *Visual Acuity*. The most surprising one is *Inspection Time*. The statistical analysis revealed that a longer inspection time did not automatically lead to a higher accuracy. This might have been caused by a poor search strategy (see Section 5.7), insecurity, task novelty or excessive demand. It is also interesting that participants could not make a reliable estimate of their own ability, i.e., their confidence is not a dependable self-judgement of their performance. The results show that self-confidence is an unreliable indicator of both inspection accuracy and inspection time. The implications of this, from the perspective of an industrial employer, are that human performance on this task needs to be measured rather than determined by self-report. Furthermore, from a training and continuous-improvement perspective, it would seem necessary to de-bias operators about their ability.

### 4.2. Inspection Time

The inspection time was measured for each participant and each blade. It represents the time from the first appearance of the image on the screen to the moment the participant advanced to the next blade. The results for each expertise group and cleanliness type are shown in Table 8. Note that *lower* inspection times are preferable from an operational perspective.

The results show that the inspection time was reduced on average by 10.4%, 3.1%, and 12.9% for inspectors, engineers, and assembly operators, respectively. On average, across all participants, clean blades led to 8.9% time savings.

#### 4.2.1. Hypothesis Testing

**Hypothesis** **H1b.** *Inspectors perform faster than non-inspecting staff.*

**Figure 5:**
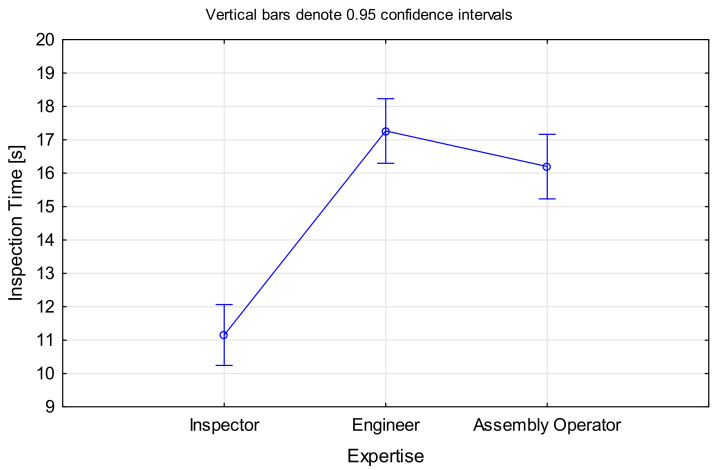
Effect of expertise on inspection time.

The hypothesis for time was analysed using ANOVA, with *Inspection Time* as the dependent variable and *Expertise* as the categorical variable. There was statistical support for this sub part of the hypothesis, F(2, 1197) = 47.238, *p* < 0.001 (Figure 5). Inspectors were on average 1.54 times faster than engineers and 1.45 times faster than assembly operators. There was no significant difference between engineers and assembly operators.

As expected, inspectors performed the task faster than non-inspecting staff. This could either be caused by a better search strategy (see Section 5.7) allowing them to find the defect faster, or the fact that they were able to make a quick decision based on their experience, while non-inspecting staff required additional time to make the final determination.

**Hypothesis** **H2b.** *Cleaned blades lead to improved inspection performance measured in inspection accuracy compared to blades in dirty condition.*

**Figure 6:**
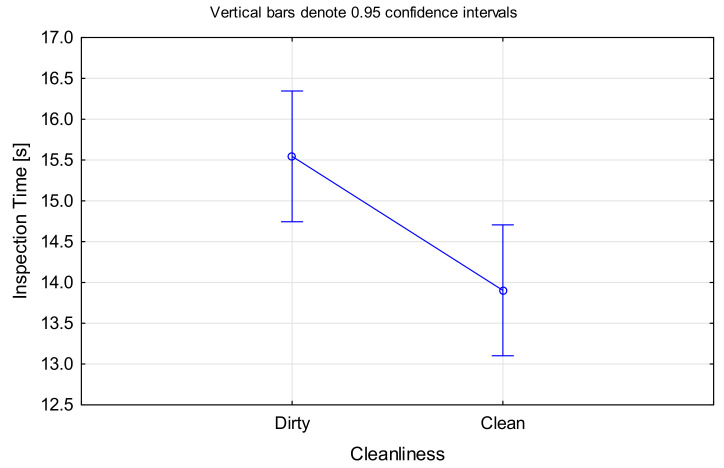
Effect of cleanliness on inspection time.

An ANOVA was performed for this hypothesis, this time with *Cleanliness* as the categorical factor and *Inspection Time* as the dependent variable. All participants performed faster on clean blades compared to dirty ones, F(1, 1198) = 8.0772, *p* < 0.005 (Figure 6). While the average time saving of approximately 1.5 s per blade does not seem much, the time adds up considering there are several hundred blades in a single engine.

Deposits are often confused with edge damage or can hide critical defects. Thus, dirty blades in particular necessitate a detailed assessment. With deposits being washed off on clean blades, the inspection was faster, as such detailed inspection is no longer required.

#### 4.2.2. Statistical Model for Inspection Time

A generalised linear/non-linear normal log model was constructed around *Inspection Time* as the dependent variable, and the following independent variables: the categorical variables were *Expertise*; *Cleanliness*; *Defect Type*; *Visual Acuity*; *Education*; *Previous Inspection Experience*; and *Decision*. The continuous variables were *Work Experience* and *Confidence Rating*. The results are presented in Table 9.

Results show that *Work Experience*, *Confidence Rating*, *Education*, *Previous Inspection Experience*, and *Decision* were not significant. Instead, the significant variables were *Expertise*, *Visual Acuity*, *Cleanliness* and *Defect Type*. *Expertise* and *Cleanliness* were discussed earlier and are not repeated here.

**Figure 7:**
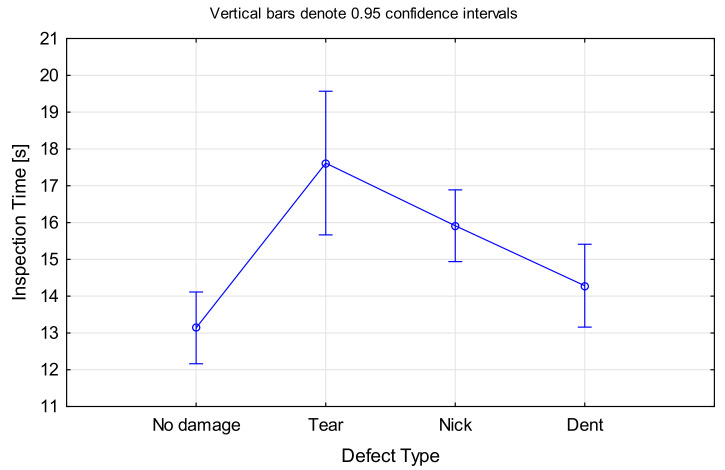
Effect of defect type on the inspection time.

In the case of *Defect Type*, the significant effect was only for tears and nicks, while the time required for dented blades was not significantly different to the one of non-defective blades, F(3, 1196) = 8.3188, *p* < 0.001 (ANOVA results in Figure 7; please note that this ignores cleanliness as a factor). Surprisingly, the inspection times for nicks and tears were longer than for dents, although the former are more salient defect types. A look at the inspection accuracy for the different defect types (as presented in Section 4.1.2) revealed that dents had a high false negative rate (often missed), which could be the reason for a shorter inspection time. However, there was no significant correlation between *Inspection Accuracy* and *Inspection Time*.

It came as a surprise that visual acuity had an effect on the inspection time, F(1, 1198) = 38.478, *p* < 0.001. Participants without glasses were on average 3.5 s faster than the ones with corrected visual acuity. This might be attributed to the study being screen-based and wearing glasses might have caused issues such as glare or reflections either on the screen or from the ceiling lights.

It is worth briefly commenting on the observation that *Inspection Accuracy* was not correlated with *Inspection Time*, F(2,1194) = 0.05997, *p* = 0.94. While the average inspection time does vary greatly with expertise (inspectors are much quicker than the other roles), within any one group, the time taken for correct vs. incorrect decisions is about the same. This implies that people looked at the images for the same length of time.

## 5. Evaluation of the Eye Tracking Data

### 5.1. Observations about the Experimental Arrangements

Given that there was no time limit and participants could inspect images at their own pace, there were no complaints about time pressure. This coincides with the findings of [27]. However, a frequently made comment was that the task was highly repetitive, and thus tedious and tiring. This emphasises the challenges in visual inspection tasks and the importance of human factors such as fatigue or complacency, which implies a high risk that can cause incorrect detections and decisions. It confirms the need to investigate those factors further and the impact they have on the inspection performance.

It was observed that during the inspection task, some participants moved and tilted their head when trying to get a better perspective of the blade. While doing so, they expressed the need to see the blade from a different perspective to get a better depth perception and to make a final decision. However, since they were presented with a static image, the head movement did not improve their view or performance. Another frequently made comment was that the participants would like to rub off the deposits with their fingers to be able to see whether the dirt was hiding any damage.

The exit survey revealed that engineers and assembly operators in particular found it challenging to detect unfamiliar defects. Moreover, they stated that it was difficult to make a decision on whether a discovery was acceptable when they were unfamiliar with the allowed defect limits. For the dirty blades, participants commonly mentioned that it was difficult to differentiate between deposits and defects, in particular those built up on the leading and trailing edge.

Several inspectors made a comment on the challenge to judge the condition of a blade based on an image, while in practice they would hold that part in their hands. Handling enables them to view the blade from different angles, and also feel it. This finding implies that—although it is called a visual inspection task—it is not purely visual, but also encompasses a tactile component, whereby the inspectors would feel along the edges for any deviations in shape and for rough or uneven surfaces. This would be an interesting area for future research and could be performed under eye tracking conditions, perhaps using wearable eye tracking glasses. Optimal viewing perspectives could be extracted from such a study.

### 5.2. Visual Search Strategies

There was a tendency across all participants, irrespective of the blades in question, to focus on inspecting the edges of the blade as opposed to the centre of the airfoil. This could be an intentional or intuitive behaviour when inspecting blades, with all recruited participants working at an engine MRO organisation and knowing that the most critical defects are defects on the edges.

**Figure 8:**
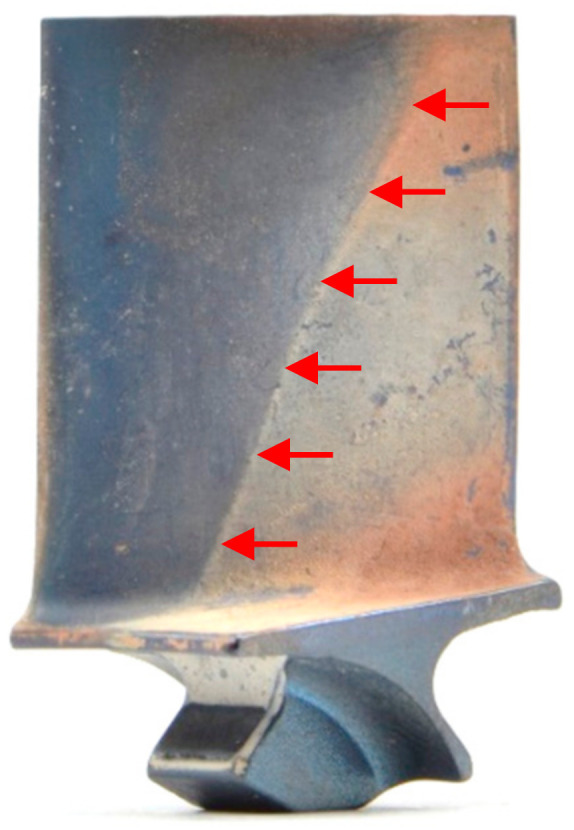
Blade with deposits forming a diagonal line that attracts the participants’ attention.

Participants tended to inspect (scan) the blade more than once to ensure that they had not missed a defect. Generally, engineers and assembly operators performed more scans than experienced inspectors, and thus needed more time for their inspection (further discussed in Section 5.7). When doing so, they may or may not have an explicit search strategy. It was found that there are common ‘paths’ they follow—mostly lines. These could be the leading or trailing edge, along the platform, the contour of the root or any striking features, such as diagonal lines formed by deposits on the airfoil (Figure 8).

### 5.3. Example of a Structured Search

**Figure 9:**
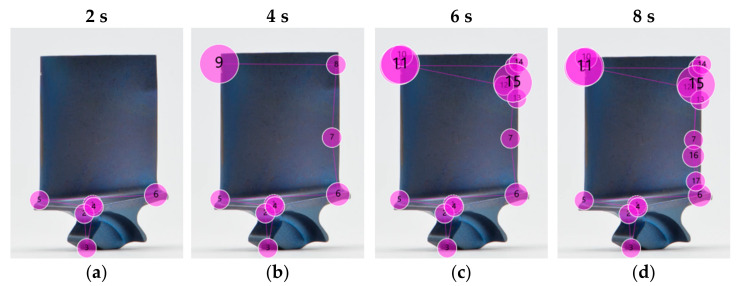
Example of a structured search shown in two-second intervals: (**a**) shows the first 2 s, (**b**) shows the first 4 s, (**c**) shows the first 6 s, and (**d**) shows the full 8 s.

An example of a structured search is shown in Figure 9. The participant started with the inspection at the corner of the platform in the centre of the image, and then moved down to the root and back up to the platform, which was inspected from left to right (Figure 9a). The left to right movement is a natural preference of most participants, possibly since it aligns to their usual reading direction [112]. In a second step (Figure 9b), the participant inspected the trailing edge and the tip of the blade until they stopped and dwelled at the location of the first defect (gaze plot number 9). The longer dwell time is visualised by the size of the gaze plot. After identifying this defect, the participant continued the search, and their eyes went back to the trailing edge and dwelled at the second defect (gaze plot 15 in Figure 9c). Gaze plots 12, 13, and 14 indicate that the neighbouring regions were inspected first before a decision was made on whether the finding is a defect or an acceptable condition. The final eye movement went down the leading edge (Figure 9d) where nothing significant was found (small gaze plots). Interestingly, the leading edge was not inspected any further (area between gaze plot 5 and 9).

This example is noteworthy, since the participant did not move their eyes straight to the obvious defect, but rather followed a structured search strategy to ensure no defect was missed.

### 5.4. Types of Inspection Errors Leading to Missed Defects

In inspection, there are three different types of errors that can occur and cause a critical defect to be missed, namely search error, recognition error and decision error. It is important to understand which type of error occurred in order to address it by appropriate means [113]. A search error, or scanning error, happens when a participant fails to look at the defective region of the blade [114,115]. This can be easily seen in the eye tracking data when there are no fixations on the defective area. If the participant failed to fixate on the defective area, then a recognition of the defect will not happen and consequently a decision cannot be made.

A recognition error in contrast arises when the participant fixates on the defective region, but the defect is not recognised as relevant enough for further consideration in the decision-making processes. One method to differentiate between successful and failed recognition of a defect is to look at the fixation durations and apply a quantitative threshold. If the fixation duration is below the threshold, it is considered as failed recognition, whereas a fixation duration above the threshold indicates a successful recognition. Common fixation thresholds range from 600 ms [116] to 1000 ms [117]. A recognition error might be caused by low prevalence and low salience [118], i.e., the defect might be rare and might not be visually prominent in terms of shape, contrast or location on the blade.

**Figure 10:**
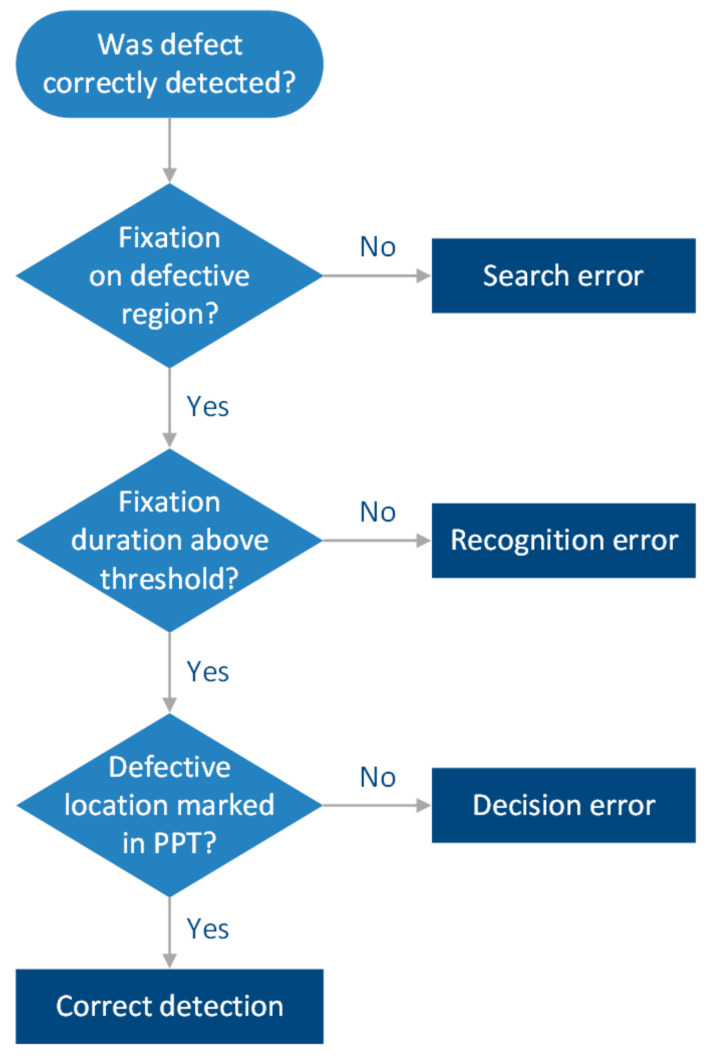
Flowchart for defining the type of error in the inspection task.

One drawback of the fixation duration method is that a longer fixation time can also be associated with difficulties in the interpretation of the finding. Thus, Brunyé et al. [114] suggested that additional evidence (e.g., think-aloud protocols) is necessary to be truly certain that a feature was successfully recognised. In this work, we introduced a stimuli presentation using Microsoft PowerPoint and the pen function thereof. This enabled us to gather the evidence needed (defect markings) to determine whether a condition was detected and classified as a defect correctly or whether it was solely looked at. Based on the eye tracking data and inspection results recorded in PowerPoint, the type of error can be determined using the flowchart in Figure 10.

A poor inspection would show a long inspection time with many gaze plots being below the threshold fixation duration. This would indicate a wandering of the eye without knowing where to look at and not perceiving any important information. The likelihood of a defect being missed in this situation is relatively high. Thus, a longer inspection time does not necessarily mean a more comprehensive inspection.

### 5.5. Improved Inspection Accuracy for Clean Blades

**Figure 11:**
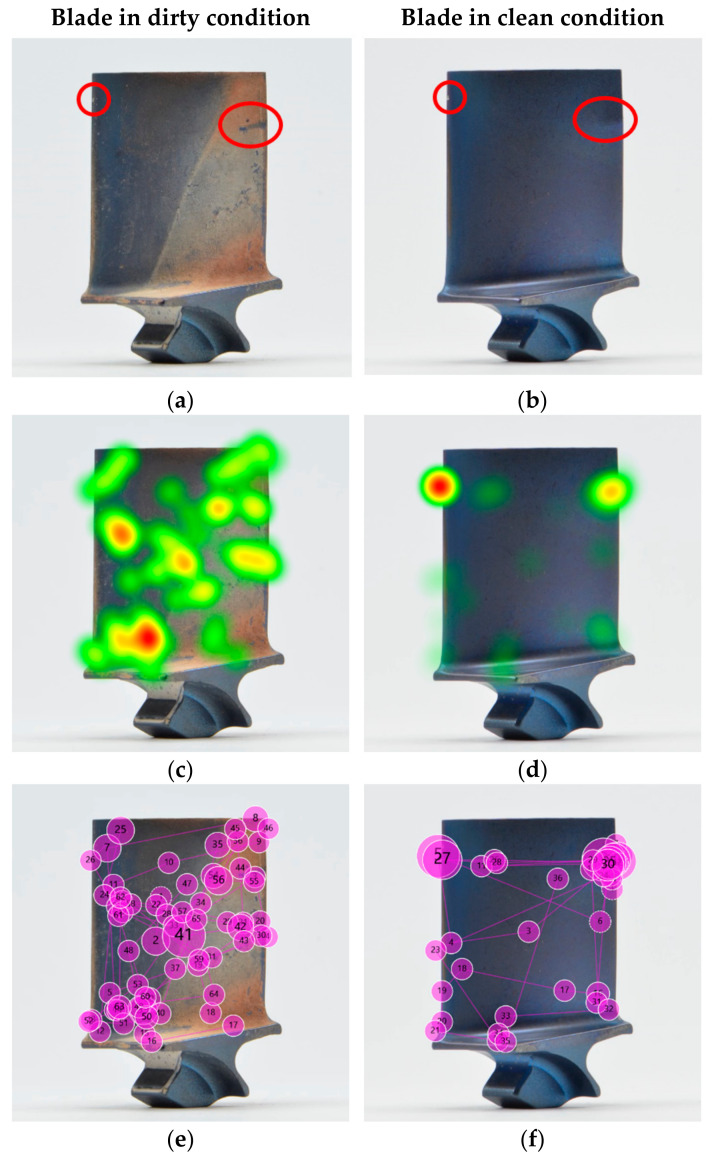
A blade with two defects (indicated by red circles) was presented to participants in (**a**) dirty and (**b**) clean condition. The heat maps (**c**,**d**) and gaze plots (**e**,**f**) were created.

The statistical analysis in Section 4 revealed that there is a correlation between inspection accuracy and cleanliness, and between inspection time and cleanliness. Next, we analysed the eye tracking data in the form of heat maps and gaze plots (Figure 11) for the same blade in dirty and clean condition to compare them and understand any variations that might explain the measured differences in inspection accuracy and inspection time.

The heat maps show a clear difference between the dirty and clean blade. The dirty blade received geometrically dispersed attention, and the heat map shows that almost the entire blade was comprehensively inspected, with exception of the root. Nonetheless, two areas stood out—one at the bottom left of the airfoil close to the platform radius and one on at the centre-left of the airfoil close to the leading edge. None of them coincided with the actual defects. In contrast, in the heat map of the cleaned blade two heat spots in warmer colour (red and orange) clearly stand out, which both align with the defective locations.

For the sample blade shown in Figure 11, the inspection time for the dirty blade was 18.094 s for 63 fixations, while the time for the cleaned blade was 11.453 s for 35 fixations. Hence, the gaze plots revealed that there were almost double the number of fixations on a dirty blade in comparison to a cleaned one, and nearly double the time. Thus, the inspection of the blade in dirty condition was 58% longer than in clean condition. The disorganised eye movement, increased fixations and longer fixation durations indicate a higher workload and decision uncertainty, and thus may imply a higher risk of missing a critical defect or making an incorrect decision. Those findings align with other research results, whereby an increase in the fixation parameters implied a higher level of complexity and cognitive load [63,114,119,120,121,122,123,124]. Hence, it can be concluded that dirty blades are more challenging to inspect.

The visualisations of the eye tracking data shown in Figure 11 are of the same participant inspecting the same blade in dirty and in clean condition. It can be seen that the crisscross search pattern did not significantly change. This indicates that the underlying search strategy is less affected by the cleanliness but rather depends on the participant. A comparison of the different search strategies is discussed in Section 5.7.

While the presented example shows the results of an assembly operator, the effect of reduced fixations and inspection time is consistent across the different levels of expertise and is further discussed in Section 5.7.

### 5.6. Decreased Inspection Accuracy for Clean Blades

Despite a general significant improvement in inspection accuracy from dirty to clean blades, in a few cases and for one blade in particular, the detection rate decreased. While eight participants (16%) performed better on the clean blade, the detectability of eleven others (22%) decreased, and the remaining 31 participants (62%) performed equally well in both conditions. Thus, participants tended to fall into two categories: the ones that were equally accurate on clean and dirty blades (N = 31), and the others that had a more exclusive preference for either clean or dirty blades (N = 19). To test this effect, the correlation between the improvement in the detectability of this particular blade and the overall improvement in detectability for all blades was analysed for each of the 19 participants. The results show that the effect of a personal preference is significant, F(1,17) = 25.702, *p* < 0.001.

**Figure 12:**
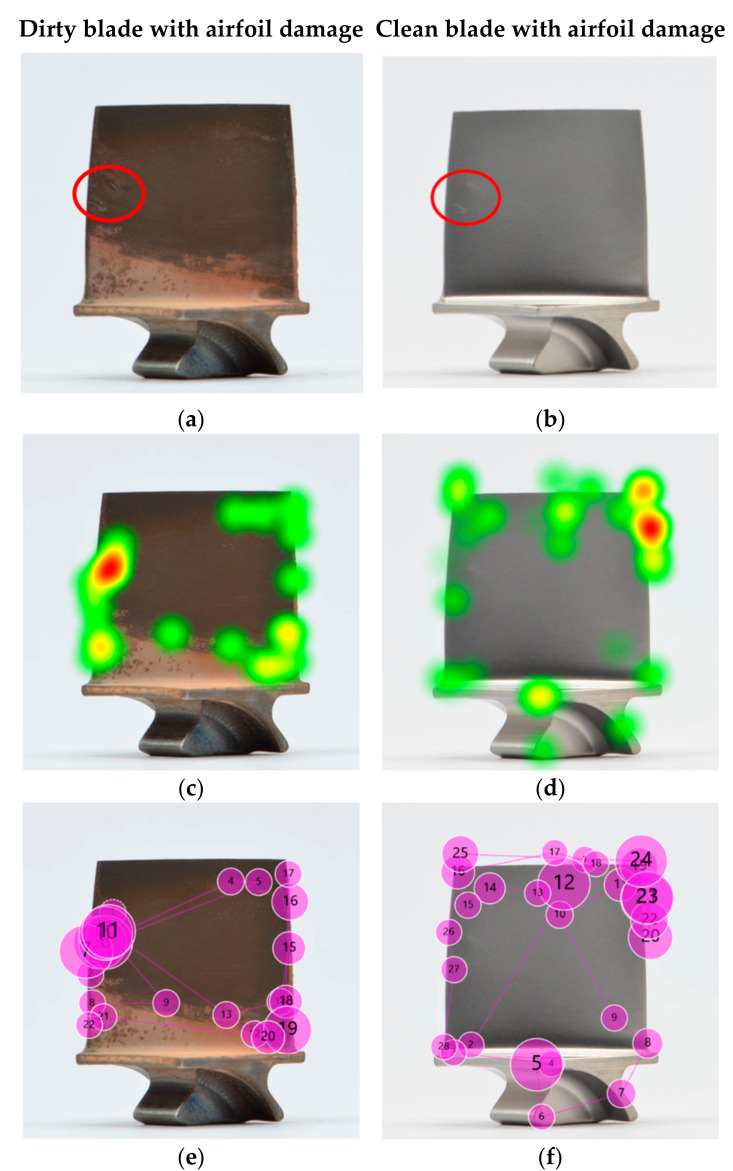
A blade with an airfoil defect (indicated by red circle) was shown to the participants in dirty (**a**) and clean (**b**) condition. The heat maps (**c**,**d**) and gaze plots (**e**,**f**) were created for an assembly operator, who detected the defect in dirty condition but missed it when the blade was cleaned.

When looking at the images of the blade in dirty and clean condition (Figure 12a,b, respectively), it becomes apparent that the airfoil defect (indicated by red circle) stands out more on the dirty blade. The higher contrast between damage and airfoil in the dirty condition might make this defect more visible and thus more likely to be detected [125]. The heat maps and gaze plots were created, and one example of an assembly operator, who detected the defect in dirty condition but missed it when the blade was cleaned, is shown in Figure 12c–f. The results revealed that the participant focused on the defect in dirty condition for quite some time, while in clean condition their eyes briefly moved over the defective location (indicated by small gaze plots). The short dwell time on the defective location could be either caused by (a) the participant making a quick decision with the outcome that the condition was not a defect, or (b) the participant not visually recognising any relevant condition that needed further investigation. The extreme short dwell times of less than 200 ms suggest that the latter might be the reason for missing the defect (recognition error). Furthermore, it seems like something on the opposite edge caught the participant’s attention. However, it remains unclear what might have caused this, since there is no salient feature visible.

### 5.7. Comparison of Search Strategies between Expertise Groups

**Figure 13:**
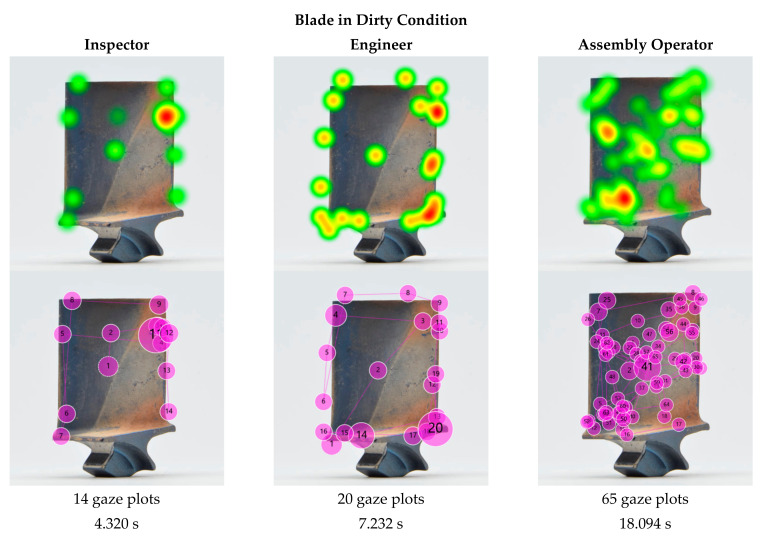
Eye tracking results for dirty blade by expertise group.

**Figure 14:**
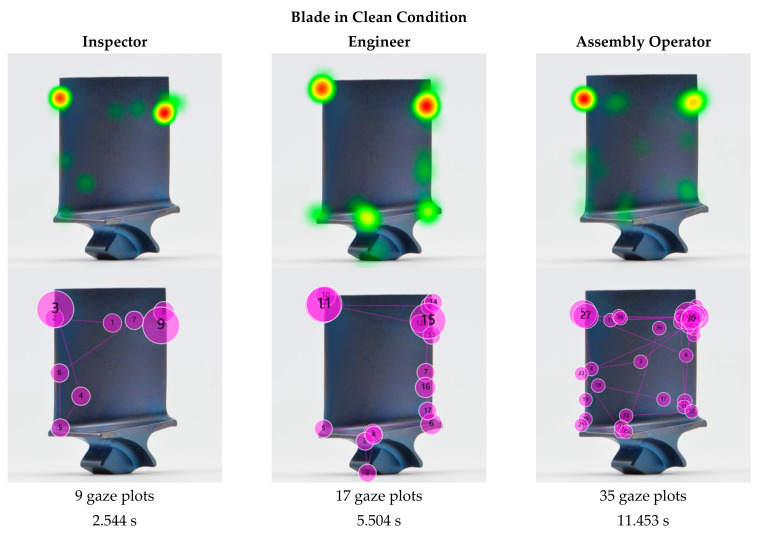
Eye tracking results of the different levels of expertise group for the same blade shown in Figure 14 but in now cleaned condition.

In the following example, we analysed the search patterns of the different expertise levels. For a fair comparison, three representative participants (one of each expertise group) were selected that had the same inspection results, i.e., each of them missed the two defects on the dirty blade (Figure 13) but found both on the clean blade (Figure 14). The participants’ eye gaze was analysed qualitatively and quantitatively to assess any deviations. The gaze plots show the trajectory of eye fixation and include both the sequence (order of observation) and a qualitative measure of the time duration (diameter of circle).

From the eye tracking data, the inspection times were extracted and compared. The comparison was made between the different expertise groups and the blade condition (dirty vs. clean). All three participants—inspector, engineer and assembly operator—performed a faster inspection on clean blades than on dirty ones, with a 41%, 24%, and 37% shorter inspection time, respectively. Furthermore, the inspection time of the inspector, engineer and assembly operator differed. The inspection of the dirty blade took the engineer 67% longer than the inspector, and the assembly operator needed two and a half times longer than the engineer and over four times longer than the inspector. The same effect was observed with the cleaned blade, whereby the assembly operator required double the amount of time than the engineer, who in turn needed twice as long as the inspector. Consequently, the assembly operator was four times slower than the inspector. Thus, although the inspection results were the same for all three participants, the study showed that there was a significant difference in inspection times. A closer look at the respective heat maps and gaze plots revealed that the participants applied different search approaches and that different inspection errors occurred.

In the dirty condition, all three participants missed the defects—but for different reasons. The heat map and gaze plot of the inspector (Figure 13) revealed that the participant did not look at the defective area on the leading edge at all (search error). The defect on the trailing edge, however (gaze plot 11), was looked at for quite some time, but it was not determined as defective and thus a decision error occurred. Both the engineer’s and assembly operator’s eyes moved over both defective areas (gaze plots 4, 10, 11, and 3, 26, 55, respectively), but failed to recognise them as defective (recognition error).

The heat maps of the clean blade (Figure 14) show that all participants found the defects and that their eyes dwelled at the defective location for quite some time. The successful detection was confirmed by their marking results. More interesting are the gaze plots, which show the participants’ search paths. The inspection expert’s eyes quickly focused on the defective area within a few fixations, even before the search process started. This phenomenon was already observed in previous studies, such as in diagnostics of medical images [126]. The researchers concluded that for experts, an initial holistic recognition of a feature preceded the detailed search and diagnosis. Novices, in contrast, were more attracted to salient features such as higher contrast, brightness or strongly pronounced geometries. However, with increasing complexity (dirty blades), inspectors tended to fall back into the same search pattern as non-inspectors and conduct a (more) structured search first.

It stood out that in both conditions, dirty and clean, the inspector’s and engineer’s search was much more structured and systematic, whereas the assembly operator scanned almost the entire blade in an unorganised and random crisscross pattern. Furthermore, the former focused predominantly on inspecting the edges, as opposed to the airfoil. While theories of visual attention [127] suggest that inspecting those areas is more likely than plain surfaces such as the airfoil, our results show that this is only true for inspectors and engineers, not for assembly operators. Thus, we would expect to see the opposite, i.e., if inspecting edges and corners is a natural behaviour, it would be more likely to be seen in the novice category as opposed to the experts. This suggests that the reason is rather related to the pre-existing knowledge and experience of the participants than to the visual regions [128], i.e., inspectors knew to look at edges, whereas novices did not. The importance of edges is explicitly developed as part of their training. The ability of experts to differentiate relevant from less relevant features, while novices consider both equally, is already discussed in educational theory [129].

Inspectors gain experience with every day and every part they inspect, covering a variety of different conditions, defect types, locations and severities. Engineers typically review the images taken by the inspectors in cases of uncertainty to confirm whether a condition is a defect. Thus, it can be concluded that both groups have a better understanding of blade defects in general. It should be noted that almost half (44%) of the engineers had previous experience in the visual inspection of blades, which might have further contributed to their pre-existing knowledge. Assembly operators, in turn, only see clean blades as part of their daily job with no deposits or defects on them (unless the part has some transportation damage, which is considerably rare). Hence, this group was considered as having the least experience.

This is also evident in the eye tracking data, whereby the heat map and gaze plot of the assembly operator stood out from the other two participants. It is apparent that the assembly operator’s search took significantly longer and required more gazes than the other two. The majority of gaze plots are found on the airfoil (centre of the blade), which indicates a poor search strategy due to missing training and previous experience [15]. Moreover, it is apparent that less experienced participants tended to revisit suspect areas (defective and non-defective) multiple times. An explanation for this behaviour might be their confidence level. When inspectors found and classified a defect, they marked it straight away and move on. This implies that they were confident with their decision made. Assembly operators in contrast returned multiple times to re-confirm that the finding was a defect, which indicates a low level of confidence in their search findings. This finding that less-experienced staff are less confident also emerges from the self-ratings of their confidence level (requested in the exit survey). The random search behaviour seems typical for novices, as they lack experience and thus confidence [36,130]. It should be noted that gaze plots, which represent the observational trajectories, may be person specific, i.e., inspection accuracy may be achieved by different trajectories.

### 5.8. Towards a Mental Model of Visual Inspection

**Figure 15:**
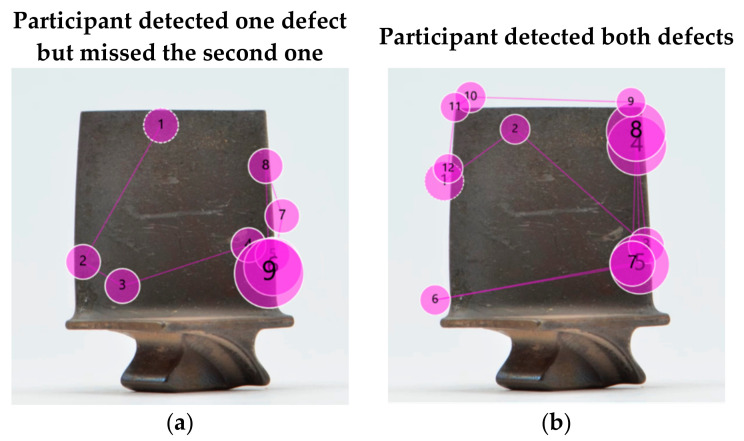
Eye tracking results of (**a**) inspector and (**b**) engineer for a compressor blade with two defects on the trailing edge.

Differently than anticipated, inspectors did not always perform best and there was no significant difference between them and the engineers and assembly operators in terms of inspection accuracy. In fact, they even performed worse in some cases. The following example of a blade with two defects is a case in point. The marking results and gaze plots of an inspector (Figure 15a) and an engineer (Figure 15b) show that both found the bigger defect (gaze plot 9 and 7, respectively). While the inspector moved on to the next blade straight away after finding the defect, the engineer in contrast continued the search on the same blade and found the second defect as well (gaze plot 8 in Figure 15b).

There are two conclusions that can be drawn from this observation. First, it comes as no surprise that a defect that is more salient in size, shape or contrast is likely to be detected first. The second finding, however, is more interesting, i.e., it was the inspector who found the bigger defect but missed the smaller one, and not the engineer. This implies that there is an underlying mental model whereby inspectors intuitively apply lean principles and do not waste time on further inspecting a blade that has already been confirmed as being defective with the finding of the first defect. This behaviour is quite common in industries where working under time pressure is the norm. The inspector does not deem additional time justifiable if the first detected defect requires the blade to be scrapped or repaired. The general literature calls this phenomenon ‘satisfaction of search’ [131].

While from a business point of view, this approach is highly efficient, it also entails the risk that the detection of highly visually salient damage can lead to the premature termination of the search and consequently cause other defects that are less salient but potentially more critical to be missed. The detection of the latter is important for the subsequent repair task. Similar observations were made in the medical industry, whereby a diagnostician prematurely made an incorrect diagnosis based on the first finding [130,131].

Another interesting finding is that none of the participants focused on the salient marking on the centre of the airfoil. This further supports the idea of an underlying mental model, whereby the knowledge that those types of markings are non-critical is stored in their subconscious mind.

## 6. Discussion

### 6.1. Summary of Work and Comparison with Other Studies

This study evaluated the effect of cleanliness and expertise on the inspection performance (accuracy and time), among other demographic parameters. First, a statistical analysis was performed, followed by a semi-qualitative evaluation of the eye tracking data.

The results show that on average, an inspection accuracy of 67% for dirty blades and 87% for clean blades was achieved. This is comparable with previous studies: in manufacturing, where parts are typically in clean condition and inspected for manufacturing defects, inspection accuracies ranged from 45% to 76% [27,29,31,95,132]. Inspections as part of maintenance procedures are typically performed on dirty parts that have already been in use, and thus intend to detect operational defects. Previous research in this area reports accuracies of 53% to 68% [4,28]. Thus, the results of this study are consistent with other findings in the general literature but are still considerably off the targeted 100% mark.

**Figure 16:**
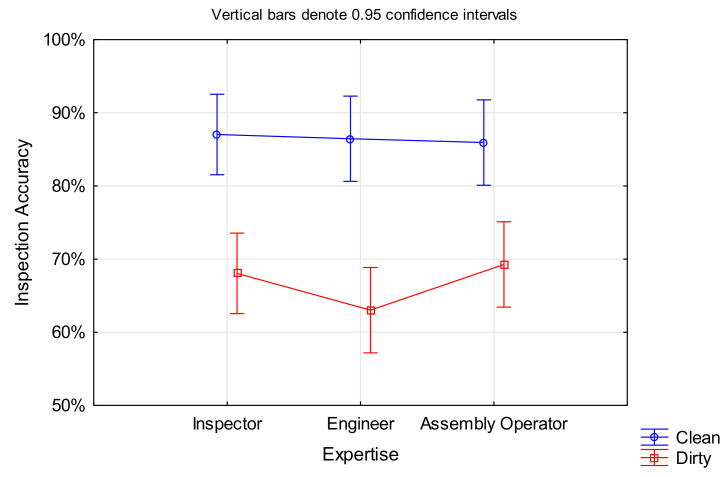
Effect of cleanliness on the inspection accuracy for each group of expertise.

The statistical analysis of inspection accuracy revealed that the part cleanliness has a significant impact for all three groups (Figure 16). Clean blades are 3.25 times more likely to be detected than dirty ones. This aligns to the findings of Baaran [24] and might be due to a reduction in salient features, such as deposits that are often confused with defects. Thus, from a safety perspective, the cleaning of blades prior to inspection is desirable. The assumption that deposit might highlight defects was generally not supported by this study.

An unanticipated finding was that there was no correlation between inspection accuracy and level of expertise, i.e., inspectors had similar accuracy performance to non-inspecting staff. The general literature is ambiguous on this. While some studies support our finding that there is no dependency [25,95,130], others found a strong correlation between expertise and accuracy [24,119]. The opposite findings could be due to different task complexities, and whether pre-existing knowledge is essential. It is possible that the contextual factors are important.

**Figure 17:**
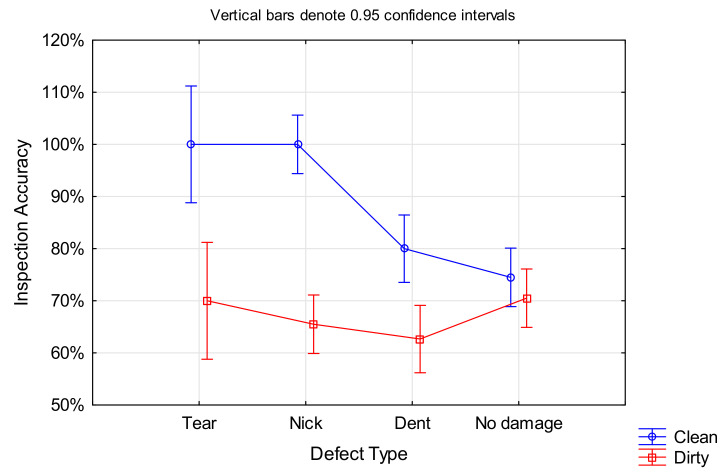
Effect of cleanliness on the inspection accuracy for clean and dirty blades.

It came as no surprise that more salient defect types such as nicks and tears led to higher detection rates, while dents were often missed. Cleanliness further improved the inspection accuracies, with the largest effect on nicks and tears, while dents showed a lower but still substantial performance improvement on clean blades. In the case of non-defective blades, however, the cleanliness made no difference (see Figure 17).

In passing, it may be worth noting that although dents are more difficult to detect compared to tears and nicks, they are also less critical from a safety standpoint, since it is unlikely that they would propagate and cause an engine failure. It is not impossible that participants applied this tacit knowledge to the task.

In accordance with previous research [27], the gaze plots showed that most participants looked at salient features first as opposed to starting the inspection at a particular location. If there was more than one defect present on a single blade, the less salient one was quite often missed. Eye tracking revealed that the search was terminated straight after the most obvious defect was found. This behaviour was found previously, but not further explained [25]. This finding emphasises the idea of an underlying mental model based on previous work experience. In practice, once a defect is found that validates the removal of the part from service, the inspector stops the inspection and continues with the next blade. This behaviour is further imposed by the inherent time pressure of the operations. Searching for additional defects would add time non-valuably, and therefore, be wasteful according to lean principles.

The comparison of findings to those of other studies [24,25,27] confirms that most demographic variables were not correlated with inspection accuracy. In this work, the non-significant parameters include work experience, previous inspection experience, education, and visual acuity. The effect of gender and age was not analysed in this study. Spencer [25] found that previous experience in inspection had no effect on the inspection performance; however, the overall work experience in the industry had indeed. This could be indirectly seen in our results and interpretation as well, in the way that all participants were industry practitioners with several years of experience and had a clear understanding of the importance of finding critical defects (underlying mental model). As the results show, there was no difference between their job roles or whether participants had previous experience in inspection.

There was statistical evidence that on average, less time was spent on clean blades compared to dirty ones. The gaze plots revealed that dirty blades had notably more fixations compared to the same blade in clean condition. The reason might be that dirty blades have more salient features that require careful consideration and more detailed inspection to make a decision on whether the feature is a defect or an acceptable condition such as a deposit. The increase in fixations explains the longer inspection times on dirty blades compared to clean ones. The exit survey showed that most participants found the inspection of dirty blades more challenging. This is backed up by the general literature that agrees that an increase in fixation parameters implies a higher task complexity and cognitive load [64,114,119,120,121,122,123,124].

**Figure 18:**
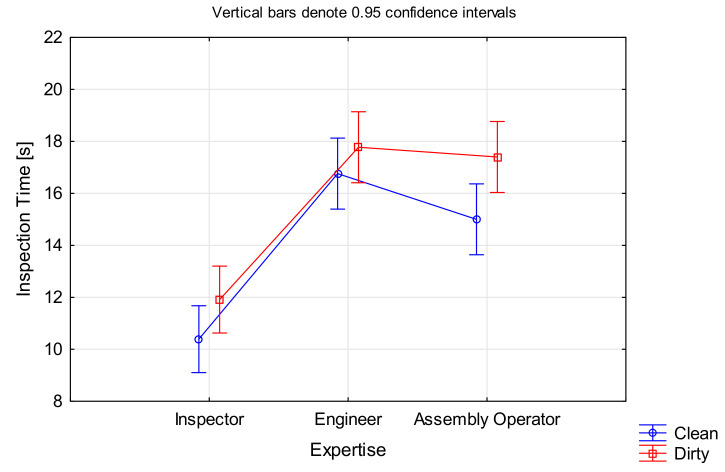
Effect of cleanliness on the inspection time for each group of expertise.

Consistent with the literature [95,130] was the finding that inspection times varied significantly between the different groups of expertise. As expected, inspectors were faster than engineers and assembly operators. No significant difference between the latter two groups was found. The gaze plots of inspectors (experts) showed the most structured search with fewer fixations and revisits thereof, leading to shorter inspection times. Engineers and assembly operators, in contrast, did not search systematically. The evaluation of their gaze paths revealed that their eyes wandered across the stimuli in a crisscross pattern between far off gaze points, causing greater distances between those. Ultimately, engineers’ and assembly operators’ eyes had to ‘travel’ more, and thus those two groups needed more time for the inspection. This was consistent for both clean and dirty blades, as shown in Section 5.7 and Figure 18 below. The findings are consistent with earlier observations, which showed that experts had a clear search pattern with fewer eye fixations and shorter viewing times than novices [25,95,130].

Surprisingly, the visual acuity of participants had an effect on their inspection time, whereby participants without glasses inspected faster than spectacle wearers. No previous work was found that has measured this effect. Most literature analysed the effect of visual acuity on the inspection accuracy as opposed to inspection time, and found that they can be positive, negative or not related, depending on the task [24,25,27].

Another interesting finding was that inspection accuracy and inspection time were not correlated. While this is supported by the research of Spencer [25], in contrast, See [27] found that longer inspection times led to higher detection rates, and Schoonard’s [95] results showed that the fastest participants performed most accurately. The contradicting findings indicate that the task and complexity might define the dependency and direction of the effect. When reviewing the eye tracking data, we found that a long inspection time can either represent a detailed search, which leads to a higher accuracy, or contrarily an unstructured, almost chaotic search, with long distances between gaze plots and many revisits thereof, but without any improvement in accuracy. Thus, the present results support both the research of See and Schoonard. However, since the measured effect in our study was in some cases positive and in some other negative, the odds ratio straddled 1, and hence was not significant.

The results also show that participants were not able to make a reliable self-assessment, as there is no correlation between their self-judgement and inspection performance (accuracy and time). These findings are somewhat surprising given the fact that other research shows that higher confidence ratings indicate increased accuracies, and that lower confidence ratings are associated with longer inspection times [27]. Given that an overall confidence rating was acquired after the task was completed, confidence ratings for individual blades could not be extracted. Measuring individual confidence ratings after each inspection might provide statistical support to the findings of See [27].

With the help of eye gaze data, it was possible to identify the type of inspection error that occurred when an incorrect inspection was made, i.e., whether it was a search, recognition or decision error. This could not be extracted from the performance data [86].

### 6.2. Implications for Practitioners

This study shows that cleaning blades prior to inspection has the potential to significantly improve the inspection accuracy. The inspection time was also shorter for clean blades than for dirty ones for all representative participants of this study (inspector, engineer and assembly operator). Thus, it is recommended that MRO providers and blade overhaul shops consider the implementation of cleaning procedures prior to inspection where feasible. Future research on reduced inspection times for clean blades could provide further justification for the cleaning procedure.

The results and insights gained from this study could serve as informative guidelines for any organisation performing inspection as part of their quality assurance system. It could change the expectations of the achievable inspection performance of staff. Knowing the limitations of visual inspection might lead to the consideration of improved visual inspection techniques including inspection aids, or the application of other NDT techniques for critical detect types where a higher detection accuracy is required to ensure a defect-free state.

Eye tracking may provide a method for competency assessment and inspection performance gaging between different inspectors within organisations and networks [127]. Another opportunity is to use eye tracking for enhanced training in the future [51,133]. Our results and other research suggest that a systematic search strategy can improve inspection performance [50,134,135,136]. Playing the eye tracking video of an experienced inspector with a desirable structured and systematic search strategy to novices or inspectors who inspect unseen parts for the first time could have a positive effect on their learning process and search strategy, leading to improved inspection performance [65,133,137].

Furthermore, eye tracking might be used to measure and record an inspection novice’s initial competence and track their improvement as they learn. It could also be used as part of a certification process, whereby a set standard for inspection accuracy and search strategy needs to be met. Typical industry practice is for staff to undergo classroom training and a theory test, followed by hands-on experience on the shop floor. However, it is up to the supervisor to decide when the trainee inspectors are ‘qualified’ to perform the task on their own, and therefore the process is highly subjective. While this qualification process only examines the basic understanding of visual inspection, it does not consider the inspection accuracy. A quantitative performance assessment using eye tracking and inspection performance measures could overcome this limitation and provide a standardised certification process that can be applied at any MRO service provider.

Visualisations of the gaze data (e.g., gaze plots) can help practitioners to understand which inspection error occurred and caused missing a critical defect. This allows one to implement or adjust training accordingly and helps to prevent the reoccurrence of those errors.

### 6.3. Limitations

There are several limitations in this study. First, participants were asked to inspect *images* of defective and non-defective parts as opposed to the *actual parts* themselves. From comments made by the subjects, they would normally hold the part and be able to study it from different orientations and feel the blade (damage) with their hands, which was not possible for photographs. Our reason for using photographs was to provide the consistence of presentation to multiple subjects. That is, when giving a dirty blade to 50 participants, the deposit on the blade would fade away after a few participants, especially when they rubbed off the deposit with their fingers during the inspection. This would have made the results incomparable. Thus, images provided the best repeatability and a fair comparison of inspection results. Additionally, some inspection tasks such as borescope inspection or detailed confirmation inspection are screen-based, which provides further justification for the chosen research design. However, we acknowledge that if the objective was to conduct individual studies, then it may be preferable to use eye tracking glasses and 3D presentation of the attention trajectory instead.

Second, the proportion of defective and non-defective parts did not represent the common operational situation, where defective blades are much rarer. One exception, however, is the inspection of FOD engines, where the number of defective blades is significantly higher. Thus, the study represents the FOD engine situation, which is less common but can still occur multiple times a year. The over-proportional amount of defective blades might have affected the ‘defect occurrence expectation’ (complacency) and inspection behaviour of the participants in yet unknown ways [138].

Third, the time to perform the inspection task was not limited, which allowed the participants to process at their own pace. In MRO operations, staff are frequently exposed to working under time pressure, which was not represented in this study. The effect of limited time could be explored in future research.

The participants of this study were recruited from one MRO facility, and hence the research population was somewhat limited. Future research could expand the population and include participants from different companies and countries to analyse any organisational, cultural or educational factors.

For organisational reasons and due to limited participant availability, the study was carried out during day and nightshifts. While we recognise the possibility that this could have influenced the inspection results, we note that in practice, inspections are also performed during the day and night time, and thus our study represents the operational environment. However, since not all three groups were working on both shifts, a statistical analysis of the effect of the working time shift was not feasible. It should be noted that all participants took the inspection task seriously and provided positive feedback throughout, stating that they enjoyed participating.

### 6.4. Challenges in Eye Tracking Technology

Eye tracking technology significantly advanced in recent years [42,139,140]. Nonetheless, there are still challenges with eye tracking, mainly in the area of data extraction, evaluation and interpretation. The data preparation and analysis were laborious and time-consuming processes that involved several manual steps, including: (a) creating and labelling custom events, (b) replaying the recording and manually setting the custom events, (c) selecting a frame from for each stimuli and linking it to a time of interest (TOI) and (d) visualising the eye tracking data by creating and customising heat maps or gaze plots.

This process had to be repeated for each stimuli and each participant—in this study, there were N = 2515 events and just as many TOIs. Since we chose a screen recording, which allowed us to use the pen function of PowerPoint for defect marking, only a few system-generated events (N = 4) and TOIs (N = 2) were generated automatically, i.e., the start and end time, as well as the duration of the recording and calibration. While the participant events (mouse clicks and key presses) were shown on the timeline, they still required manual labelling and logging. This is a fundamental limitation of the eye tracking hardware and software.

**Figure 19:**
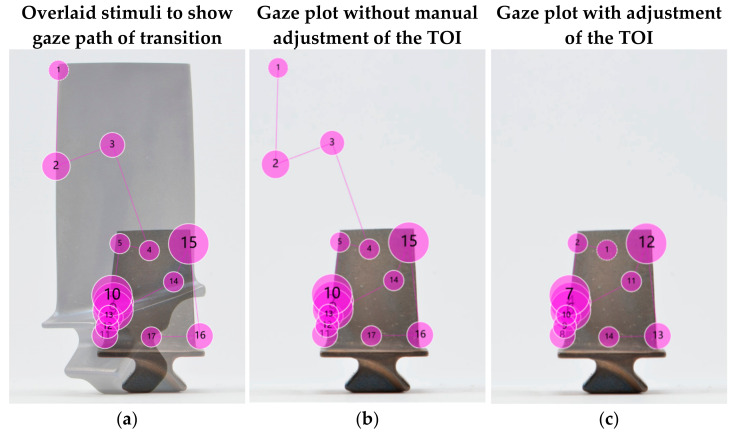
(**a**) Overlaid previous and next stimulus to show the transition of gaze plots from one to another. Gaze plot (**b**) without and (**c**) with manual adjustment of the TOI. Note that there was no fading between stimuli (as may appear in (**a**)). The opacity was adjusted manually for this paper to show both blades in one image.

When the stimulus size varied (Figure 19a), there was an unexpected side effect that needed to be taken into account, i.e., the human eye had to re-adjust to the new stimulus size. The gazes and time required to readjust should not be included in the analysis. This side effect only became apparent when we created the heat maps and gaze plots. Without manual adjustment (Figure 19b), the last gazes from the previous stimuli showed up on the next one. This problem was solved by manually adjusting TOIs and creating an offset (result shown in Figure 19c). Again, this was a time-consuming and onerous task, showing that even automatically generated time marks required manual adjustment. While it was relatively simple to exclude the relevant gaze plots in our study (due to the plain background), it would have been more challenging in situations where the stimuli showed complex environments, such as the ones shown in [64].

Not only was the eye tracking analysis a laborious task, but also it was challenging to keep track of the many markers and time stamps. A clear labelling strategy and well-organised structure was required to keep track of all the eye tracking data (markers, events, TOIs, heat maps, gaze plots, etc.), which are stored for all participants in the same project file. As previous researchers have already pointed out, there is no automated process or algorithm for the stimuli processing and eye tracking data analysis or interpretation, which is one of the largest drawbacks of eye tracking [141]. This offers great potential for future development and could make eye tracking more attractive to researchers and industry practitioners.

It was found that it was difficult to compare different heat maps and gaze plots quantitatively with each other to make a statement as to whether two search patterns were similar or not. Each gaze plot and heat map was unique and differed from one participant to another. Currently, the only possible option is the use of areas of interest (AOI) [142]. Those are particularly helpful for stimuli with multiple objects or areas that are clearly separated, such as different elements of a website or user interface. The different gaze paths might be compared based on the order the participants viewed the AOIs. In the case of, e.g., three AOIs, there are 15 possible viewing orders—providing that an AOI was not visited multiple times, otherwise the number of combinations would be infinite. While this might work for a small number of AOIs, it is difficult to apply to stimuli that have no clearly separable areas or stimuli with a large number of AOIs. The possible combinations increase exponentially with the increasing number of AOIs, which significantly reduces the likelihood of multiple participants having the exact same gaze order. The problem of comparing gaze plots and heat maps quantitatively has not yet been adequately solved.

Eye tracking technology is not yet able to collect every participant’s eye movement effectively. In N = 4 cases (8%), the eye tracker was unable to record the participants eye gaze and eye recognition rates of only 40% or less were achieved. In one case, the eye tracker failed to detect the participant’s eyes entirely (during the calibration) and thus the participant could not participate. This limitation has to be considered in the sampling phase and when recruiting participants. Pernice and Nielsen [106] suggested adding a 23% fallout rate to account for any data loss due to failed eye recognition or calibration. However, this remains particularly challenging, when only a limited amount of people, e.g., experts in the field are available.

It is not within the capability of eye tracking to tell the researcher with certainty whether the participant looked at something consciously or without any awareness. This is when someone is ‘staring into space’ and lets their gaze wander without focusing on anything particular. While this eye movement is recorded by the eye tracker, the technology cannot differentiate between actively looking and wandering. Furthermore, peripheral vision is not captured by eye tracking [143].

Eye tracking cannot explain why a participant looked at something. This reasoning is up to the researcher and requires a combination of contextual knowledge of the task, and understanding of the human vision, eye anatomy, visual perception, cognitive processing and eye physiology [144].

Although eye tracking technology advanced significantly in the past years, recent studies [145] show that the recording of the eye gaze is still very sensitive to head movement and orientation. Eye tracking manufacturers claim that the quality of the collected data is not affected by the head position, movement and orientation as long as the eyes are within a recommended recordable area (headbox) and that high accuracy, precision and tracking robustness is maintained independently [146]. Further, the manufacturer states that chin rests are only required for e.g., micro-saccade studies [147]. However, research suggests that significant precision, accuracy and data loss occurs if the head is in a non-optimal position or orientation [145]. In our study, participants claimed that it was challenging to hold their heads in a fixed position for the duration of the study, even when they were allowed some head movement (within the headbox). Neck pain was a common complaint made.

Furthermore, participants found it challenging and tiring to look at the screen for the duration of the study—particularly participants that did not work in front of a monitor in their daily job. While this problem is not specifically related to eye tracking but any screen-based study, it should certainly be considered when designing the study, e.g., by including frequent breaks. The disadvantage of frequent breaks during eye tracking studies is the need for a recalibration after each break [106].

### 6.5. Future Work

Several avenues for future research have been identified in the text above and are not repeated here. The present study provided a self-paced task, and it could be interesting to repeat the study but limit the available inspection time per blade. A difference in detection accuracy would be expected between the three groups. Lower accuracies may accompany shorter inspection times [148]. Another line of research could be to examine saccades, blink rates, and pupil diameter. Tactile vs. visual inspection tasks could also be valuable.

From an operational perspective, the detection accuracy itself is not a good measure if the inspection takes exceptionally long, and would cause a bottleneck in the production. Conversely, a fast inspection time might also be a poor measure for the inspector performance, since a short inspection time could lead to poor accuracies. Future work could focus on calculating productivity based on inspection time and accuracy. Potentially, the risk component could also be considered, i.e., defects that impose a higher risk of propagating before the next engine shop visit.

There are several concepts that could be applied to automate the visual inspection process in several ways. This could include personalised training based on the initial eye tracking results and inspection competency to accelerate the transition from novice to expert [149,150,151], monitoring of the visual expertise development [130,152], utilising the eye tracking data to train an artificial intelligence system for automated defect detection adopting the human’s search strategy and decision making [153,154], and a pre-warning system indicating when an inspection error emerges during the search, recognition, or decision-making process [155,156,157].

There is a need to support human decision making during inspection [100,158,159]. The challenge is integrating human operators and inspection software.

## 7. Conclusions

This work makes several novel contributions. First, the inspection performance of metal parts with complex geometries, namely engine blades, was measured in the form of inspection accuracy and inspection time. This included the assessment of operational defects caused by foreign object damage (FOD), including dents, nicks, and tears, but also non-defective blades. Findings were based on images of parts, rather than the physical parts themselves, and hence the tactile dimension of inspection was excluded from this study.

Secondly, the correlation between the cleanliness factor and the inspection results was statistically and qualitatively analysed. While the findings regarding cleanliness may seem self-evident, this is less obvious for industrial practitioners. Within the industry, there are two prevalent schools of thought: (a) that a compressor wash prior to inspection washes away all the evidence of the defect; (b) that deposits hide the defects, and hence cleaning is valuable. This is a complex problem because of the variety of defect types. This work makes a novel contribution by identifying the importance of cleanliness at least for nicks, dents, and tears.

Moreover, the effect of self-confidence rating and other demographic variables on the inspection time and accuracy was analysed. Additionally, eye tracking was used to extract the search strategies for different levels of expertise and part cleanliness using heat maps and gaze plots. This provided insights into the underlying cognitive and attentional processes. Overall, the present study adds to the understanding of inspection accuracies and search strategies of complex manufactured parts, not only in aviation, but the manufacturing and maintenance industry as a whole. Eye tracking methods have the potential to assist in the training of human operators for inspection tasks, and support the continuous improvement of the processes.

## Figures and Tables

**Table 1:** Demographics of participating research population (N = 50).

	Inspectors (N = 18)	Engineers (N = 16)	Assembly Operator (N = 16)
**Gender**			
Male	17	15	15
Female	1	1	1
**Corrected vision**	10	10	5
**Currently assigned to inspection**	17	0	0
**Previously been assigned to inspection (how many years ago)**			
Never	-	9	12
1 to 4	Currently	0	1
5 to 10	Currently	1	2
10 to 20	Currently	6	0
20 and more	Currently	0	1
**Work experience in the field in years**			
1 to 4	2	0	3
5 to 9	2	0	3
10 to 19	6	3	8
20 and more	8	13	2
**Highest qualification**			
Trade certificate	10	3	13
Diploma	3	6	1
University degree	5	7	2

**Table 2:** Research hypotheses.

Hypothesis	
H1	Inspectors perform better in terms of (a) inspection accuracy and (b) inspection time than non-inspecting staff.
H2	Cleaned blades lead to improved inspection performance measured in (a) inspection accuracy and (b) inspection time, compared to blades in dirty condition.

**Table 3:** Metrics to evaluate the inspection performance.

Metrics	Description
Decision	Determination whether a part is defective or non-defective. Takes the value of one (1) when a correct decision was made and zero (0) when an incorrect decision was made.
True Positive (TP)	Blade was correctly identified as defective (hit).
False Negative (FN)	Blade was incorrectly identified as non-defective and thus the defect was missed (miss).
False Positive (FP)	A non-defective blade was incorrectly classified as defective (false alarm).
True Negative (TN)	A non-defective blade was correctly classified as non-defective (correct acceptance).
Inspection Accuracy (IA)	Measure for the inspection performance taking into account the correct decisions (TP and TN), and the total population; also referred to as Decision Accuracy.
Improvement Rate (IR)	Inspection accuracy improvement between dirty and clean condition.
Inspection Time (IT)	Time needed to inspect a blade.
Confidence Rating (CR)	Self-rate confidence level of participant on a scale from one to five.

**Table 4:** Inspection accuracies by expertise group and cleanliness (in percentages).

Expertise	Dirty Blades M (SD)	Clean Blades M (SD)	Improvement M (SD)
Inspectors (N = 18)	68.1 (13.8)	87.0 (10.4)	33.6 (33.1)
Engineers (N = 16)	63.0 (10.5)	87.0 (8.03)	42.5 (32.8)
Assembly Ops. (N = 16)	69.3 (10.4)	86.5 (11.3)	29.0 (32.8)
All participants (N = 50)	66.8 (11.8)	86.8 (9.83)	35.0 (32.7)

**Table 5:** Statistical analysis of the effect of expertise on the inspection accuracy (parameter estimates and odds ratios). The probability value (*p*) is of the odds ratio.

Effect	Reference Level	Level of Effect	Estimate	Wald. Stat	Odds Ratio	Lower CL 95%	Upper CL 95%	*p*
Expertise	Assembly Operator	Engineer	−0.104208	1.1711	0.853871	0.612345	1.190663	0.279170
Expertise	Assembly Operator	Inspector	0.050441	0.2794	0.996679	0.716831	1.385778	0.597094

Parameter estimates and odds ratios. Distribution: BINOMIAL. Link function: LOGIT. Modelled probability that Decision = 1.

**Table 6:** Statistical analysis of the effect of cleanliness on the inspection accuracy (parameter estimates and odds ratios). The probability value (*p*) is of the odds ratio.

Effect	Reference Level	Level of Effect	Estimate	Wald. Stat	Odds Ratio	Lower CL 95%	Upper CL 95%	*p*
Cleanliness	Dirty	Clean	0.578399	61.4089	3.179736	2.380880	4.246631	0.000000

Parameter estimates and odds ratios. Distribution: BINOMIAL. Link function: LOGIT. Modelled probability that Decision = 1.

**Table 7:** Statistical model around the inspection accuracy (Wald test and odds ratios). The probability value (*p*) is of the odds ratio. Significant factors are indicated by an asterisk *.

Effect	Reference Level	Level of Effect	Wald. Stat	Odds Ratio	Lower CL 95%	Upper CL 95%	*p*
Work Experience			0.23356	0.994938	0.974668	1.015629	0.628900
Inspection Time			0.10953	0.997451	0.982491	1.012640	0.740683
Confidence Rating			0.62841	1.087622	0.883665	1.338654	0.427940
Expertise	Assembly Operator	Engineer	0.53783	1.167187	0.758731	1.795531	0.477357
Expertise	Assembly Operator	Inspector		1.036288	0.667100	1.609792	0.832337
Visual Acuity	No glasses	Glasses	1.40159	0.821929	0.594074	1.137178	0.236457
Education	Trade Cert.	Diploma	1.22683	1.214756	0.802685	1.838372	0.615918
Education	Trade Cert.	Bachelor		1.210193	0.804527	1.820406	0.631881
Prev. Inspection Experience	No	Yes	0.23881	0.901183	0.593719	1.367870	0.625066
Cleanliness *	Dirty	Clean	62.72209	0.305089	0.227422	0.409279	0.000000
Defect Type *	No damage	Tear	20.75906	0.451933	0.245067	0.833419	0.041402
Defect Type *	No damage	Nick		0.533789	0.375063	0.759687	0.028675
Defect Type *	No damage	Dent		1.068135	0.755904	1.509335	0.001932

Test of all effects and odds ratios. Distribution: BINOMIAL. Link function: LOGIT. Modelled probability that Decision = 1.

**Table 8:** Inspection times by expertise group and cleanliness type (in seconds).

Expertise	Dirty Blades M (SD)	Clean Blades M (SD)	Time Savings M (SD)
Inspectors (N = 18)	11.914 (4.302)	10.390 (3.827)	1.524 (2.263)
Engineers (N = 16)	17.773 (7.315)	16.757 (6.124)	1.016 (3.632)
Assembly Ops (N = 16)	17.400 (8.341)	15.001 (7.944)	2.399 (2.723)
All participants (N = 50)	15.545 (7.189)	13.903 (6.593)	1.641 (2.899)

**Table 9:** Statistical model around the inspection time (Wald test and parameter estimates). The probability value (*p*) is of the odds ratio. Significant factors are indicated by an asterisk *.

Effect	Reference Level	Level of Effect	Wald. Stat	Estimate	Lower CL 95%	Upper CL 95%	*p*
Work Experience			0.2720	−0.001457	−0.006932	0.004018	0.601996
Confidence Rating			0.0786	−0.007156	−0.057186	0.042874	0.779215
Expertise *	Assembly Operator	Engineer	11.1835	0.104217	0.043137	0.165298	0.000825
Expertise *	Assembly Operator	Inspector	69.1614	−0.286814	−0.354409	−0.219218	0.000000
Visual Acuity *	No glasses	Glasses	56.4609	0.154085	0.113893	0.194276	0.000000
Education	Trade Cert.	Diploma	2.3705	0.051595	−0.014086	0.117275	0.123651
Education	Trade Cert.	Bachelor	0.0172	0.004301	−0.059895	0.068497	0.895535
Prev. Inspection Experience	No	Yes	0.0072	−0.002031	−0.049062	0.045001	0.932561
Cleanliness *	Dirty	Clean	11.5048	−0.061458	−0.096971	−0.025945	0.000694
Defect Type *	No damage	Tear	13.0585	0.148160	0.067801	0.228518	0.000302
Defect Type *	No damage	Nick	4.2438	0.056915	0.002765	0.111064	0.039393
Defect Type	No damage	Dent	3.3302	−0.058704	−0.121753	0.004345	0.068017
Decision	0	1	1.1045	0.023340	−0.020188	0.066868	0.293284

Test of all effects and parameter estimates. Distribution: NORMAL, Link function: LOG.

## Data Availability

The data are not publicly available due to commercial sensitivity and data privacy.
